# Adverse Events and Tolerability of Combined Durvalumab and Tremelimumab versus Durvalumab Alone in Solid Cancers: A Systematic Review and Meta-Analysis

**DOI:** 10.3390/biomedicines10051101

**Published:** 2022-05-10

**Authors:** Omar Fahmy, Osama A. A. Ahmed, Mohd Ghani Khairul-Asri, Nabil A. Alhakamy, Waleed S. Alharbi, Usama A. Fahmy, Mohamed A. El-Moselhy, Claudia G. Fresta, Giuseppe Caruso, Filippo Caraci

**Affiliations:** 1 Department of Urology, Universiti Putra Malaysia, Selangor 43400, Malaysia; omarfahmy.ahmed@upm.edu.my (O.F.); khairulasri@upm.edu.my (M.G.K.-A.); 2 Department of Pharmaceutics, Faculty of Pharmacy, King Abdulaziz University, Jeddah 21589, Saudi Arabia; oaahmed@kau.edu.sa (O.A.A.A.); nalhakamy@kau.edu.sa (N.A.A.); wsmalharbi@kau.edu.sa (W.S.A.); uahmedkauedu.sa@kau.edu.sa (U.A.F.); 3 Advanced Drug Delivery Research Group, Faculty of Pharmacy, King Abdulaziz University, Jeddah 21589, Saudi Arabia; 4 Center of Excellence for Drug Research and Pharmaceutical Industries, King Abdulaziz University, Jeddah 21589, Saudi Arabia; 5 Mohamed Saeed Tamer Chair for Pharmaceutical Industries, King Abdulaziz University, Jeddah 21589, Saudi Arabia; 6 Clinical Pharmacy and Pharmacology Department, Ibn Sina National College for Medical Studies, Jeddah 21589, Saudi Arabia; m_moselhy64@yahoo.com; 7 Department of Pharmacology and Toxicology, Faculty of Pharmacy, Minia University, Minia 61519, Egypt; 8 Department of Drug and Health Sciences, University of Catania, 95125 Catania, Italy; forclaudiafresta@gmail.com (C.G.F.); fcaraci@unict.it (F.C.); 9 Unit of Neuropharmacology and Translational Neurosciences, Oasi Research Institute-IRCCS, 94018 Troina, Italy

**Keywords:** durvalumab, tremelimumab, combined therapy, monotherapy, checkpoint inhibitors, adverse effects

## Abstract

Background: Recently, the combination of durvalumab and tremelimumab, two immune checkpoint inhibitors, for the treatment of different types of cancers has been considered; however, its overall effects, including its safety, are still unclear and need to be further investigated. Objectives: The aim of the present systematic review and meta-analysis was to investigate the safety and tolerability of this combination of drugs. Methods: A systematic review of the literature, based on the Preferred Reporting Items for Systematic Reviews and Meta-analyses (PRISMA) statement, was conducted by employing online electronic databases and the American Society of Clinical Oncology (ASCO) Meeting Library. The selection of eligible publications was made following a staged screening and selection process. The software RevMan 5.4 was used to run the quantitative analysis and forest plots, while the Cochrane tool was employed for risk of bias assessment. Results: From the retrieved 157 results, 9 randomized controlled trials involving 3060 patients were included. By comparing the combination of durvalumab and tremelimumab vs. durvalumab monotherapy, it was observed that: adverse events (AEs) ≥ Grade 3 incidence was 32.6% (536/1646) vs. 23.8% (336/1414) (Z = 2.80; *p* = 0.005; risk ratio (RR) = 1.44), reduced appetite incidence was 10.8% (154/1427) vs. 8.3% (108/1305) (Z = 2.26; *p* = 0.02; RR = 1.31), diarrhea was reported in 15.6% (229/1473) vs. 8.1% (110/1352) (Z = 5.90; *p* < 0.00001; RR = 1.91), rash incidence was equal to 11.1% (160/1441) vs. 6.5% (86/1320) (Z = 4.35; *p* <0.0001; RR = 1.75), pruritis was 13.6% (201/1473) vs. 7.7% (104/1352) (Z = 5.35; *p* < 0.00001; RR = 1.83), fever was 10.5% (42/399) vs. 6.6% (22/330) (Z = 2.27; *p* = 0.02; RR = 1.77), discontinuation rate was 18% (91/504) vs. 3% (36/434) (Z = 4.78; *p* < 0.00001; RR = 2.41), and death rate was 2.6% (13/504) vs. 0.7% (3/434) (Z = 1.90; *p* = 0.06; RR = 2.77). Conclusions: It was observed that the combined (durvalumab and tremelimumab) vs. monotherapy (durvalumab) is associated with a higher risk of treatment discontinuation, mortality, fever, diarrhea, rash, pruritis, and reduced appetite. This information is relevant and should be disclosed, especially to patients that are currently enrolled in clinical trials considering this combined therapy.

## 1. Introduction

A new era in cancer therapy has been started after the introduction of immune checkpoint inhibitors (ICIs), representing the most important development in this field over the past decade [1,2,3]. These innovative drugs have shown promising results, preventing tumor immune escape through immune checkpoints, thus enabling immune cells to maintain their killing effect on malignant cells [4]. Durvalumab is a human immunoglobulin G1 kappa monoclonal antibody and a novel ICI used for cancer treatment [5]. It is a programmed death-ligand 1 (PD-L1) inhibitor able to enhance basal immune responses against tumor cells [6]. Durvalumab was granted accelerated approval by the U.S. Food and Drug Administration (FDA) in 2017 for the treatment of locally advanced or metastatic urothelial carcinoma [7]. One year later (2018), it gained another approval for the treatment of selected patients with locally advanced, unresectable non-small cell lung cancer (NSCLC) [8]. In March 2020, durvalumab was approved to be used as a first line in combination with chemotherapy for patients suffering from extensive stage small cell lung cancer (ES-SCLC) [9]. Tremelimumab is a fully humanized, anti-cytotoxic T-lymphocyte-associated protein 4 (CTLA-4) IgG2 monoclonal antibody [10]. CTLA-4, also known as CD152 (cluster of differentiation 152), is a transmembrane receptor constitutively expressed in regulatory T cells, which promotes immunosuppression in the tumor microenvironment [11]. It is an inhibitory molecule able to regulate T cell expansion and differentiation; in particular, its inhibitory activity has been related to the binding of B7-1/B7-2 ligands [12,13]. In addition to the above, it represents a negative regulator of the immune response and a target for cancer therapy [14]. Blocking of CTLA-4 with tremelimumab allows T cells to proliferate and attack tumor cells. Tremelimumab has been tested on different cancer types, including mesothelioma, bladder cancer, lung cancer, melanoma, liver cancer, and head and neck cancer, but despite some promising results, it has not yet been approved by the FDA to treat any cancer or disease [15].

Although ICIs have been used as immunotherapy to treat many kinds of cancer, leading in some patients to long-lasting remissions, they can cause a range of long-term side effects [11]. In particular, it has been strongly suggested as ICIs possess both direct or indirect reactive oxygen species (ROS)-dependent mechanisms coming from the interactions occurring between programmed cell death-1 (PD-1) antibodies and ROS generation [16], leading to a well-known phenomenon known as oxidative stress [17,18].

The use of ICIs has been associated with a higher incidence of immune-related adverse events (irAEs) compared with chemotherapy [19], often leading to irAEs that are distinctly different from the classical chemotherapy-related toxicities [11]. Common irAEs include dermatologic irAEs (pruritus and rash), endocrine irAEs (hypothyroidism and hyperthyroidism), colitis, pneumonitis, and hepatitis [20]. These irAEs, if not properly treated, might cause treatment termination, failure, or even be life-threatening and fatal [21].

The combination therapy of durvalumab with tremelimumab might boost the anticancer immune activity as each drug possesses a specific pharmacodynamics profile and a defined molecular mechanism of action; indeed, different clinical trials have investigated the combination of these drugs and have shown conflicting results regarding the side effects of adding tremelimumab to durvalumab. For instance, rash, anemia, neutrophilia, fatigue, dyspnea, asthenia, and thyroid dysfunction. All these side effects have been reported to be higher with monotherapy in some studies or higher with combination therapy in other studies [22,23,24,25,26,27]. As safety and tolerability are crucial in medical therapy, especially when employing combination protocols, the aim of this systematic review and meta-analysis was to assess the safety profile as well as the risk of increased side effects due to the combination therapy of durvalumab with tremelimumab compared with durvalumab monotherapy in cancer patients.

## 2. Materials and Methods

### 2.1. Search Strategy

We conducted an online systemic search through online electronic databases (PubMed, EMBASE, Wiley Online Library, and Cochrane databases) based on Preferred Reporting Items for Systematic Reviews and Meta-analyses (PRISMA) criteria [28,29]. The following keywords were used during the search: durvalumab, tremelimumab, immunotherapy, and checkpoint inhibitors. The exclusion criteria considered were: (1) review articles, (2) case reports, (3) letters to editors and editorial comments, (4) repeated publications, (5) non-controlled trials, and (6) clinical trial protocols. All the obtained results, initially assessed by the title, with or without abstract assessment, were followed by full-text assessment. The manual search in reference lists of relevant published studies was conducted in order to avoid missing any eligible studies. Eventually, we included the controlled trials with two cohorts, one for durvalumab plus tremelimumab, and one for durvalumab alone. For trials with multiple arms, we included only the two targeted groups (durvalumab plus tremelimumab and durvalumab).

### 2.2. Data Extraction

Data were independently extracted by two authors and checked by a third one. Discrepancies were resolved after discussion among the three authors. Extracted data: main author and year of publication, timeframe of the study, type of the study and registration number, type of cancer treated, total number of patients in each cohort, doses of durvalumab and tremelimumab, additional treatments, adverse events (AEs), and conclusion of the study. Dichotomous data for analysis were extracted as events and total numbers. The effect measurement was calculated using pooled risk ratio (RR) and 95% confidence interval (CI).

### 2.3. Primary Outcomes

The primary outcome of this systematic review and meta-analysis was to compare the side effects of the combination of durvalumab and tremelimumab vs. durvalumab monotherapy. The following grading was used for the AEs: Grade (G)1 = mild; G2 = moderate; G3 = severe; G4 = life-threatening; and G5 = death.

### 2.4. Statistical Analysis

The Nordic Cochrane Centre (Cochrane Collaboration, Copenhagen) employed Review Manager (RevMan) software version 5.4 for statistical analysis and the creation of forest plots for this meta-analysis. In each analysis, the I^2^ value was used to determine the heterogeneity among the studies. In the cases of I^2^ < 50% and I^2^ ≥ 50%, fixed and random effect models were used, respectively. The Z-test was employed to assess the overall impact. Only *p*-values < 0.05 were considered statistically significant.

### 2.5. Risk of Bias Assessment

All included studies were randomized, and the Cochrane bias assessment tool of The Nordic Cochrane Centre (Cochrane Collaboration, Copenhagen) Review Manager (RevMan), software version 5.4, was used for the assessment of the risk of bias.

## 3. Results

### 3.1. Search Results

**Figure 1:**
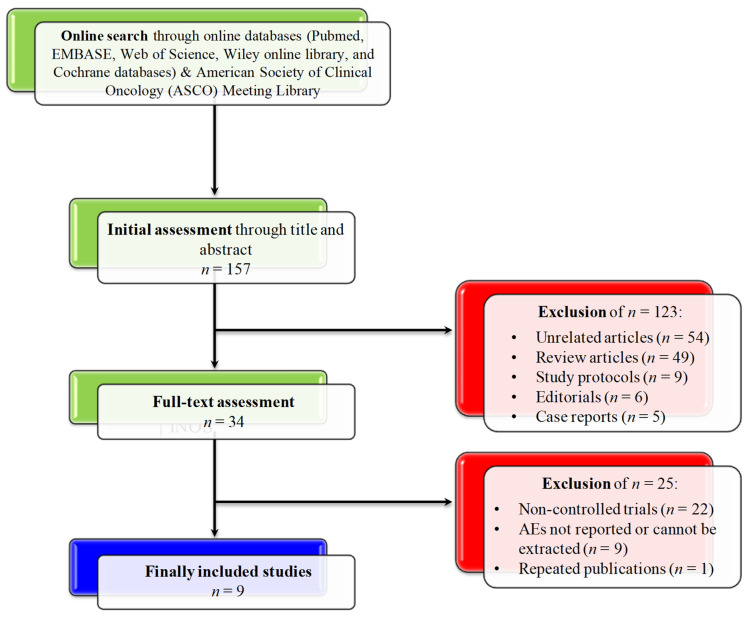
CONSORT diagram for the screening and selection processes of the included studies.

An initial search in electronic databases revealed 157 results that underwent initial assessment (through title and abstract). After this stage, 34 publications underwent full-text assessment, 9 of which were randomized controlled trials and were included in the study [22,23,24,25,26,27,30,31,32]. A total number of 3060 patients were included in the pooled analyses: 1646 (53.8%) received durvalumab and tremelimumab, while 1414 (46.2%) received durvalumab monotherapy. The flow of screening, as well as the selection process, are described in Figure 1.

A summary of the included studies is provided in Table 1.

**Figure 2:**
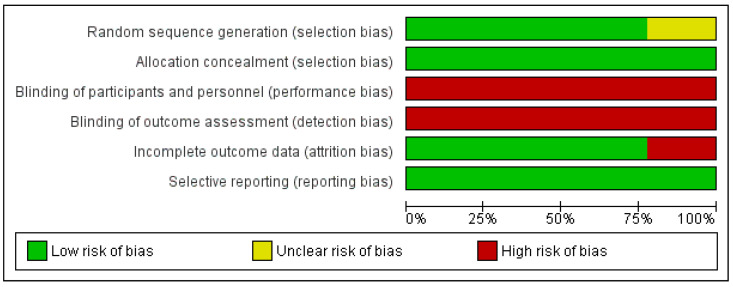
Total percentage risk of bias for all the randomized trials: green, low risk; yellow, unclear; red, high risk.

**Figure 3:**
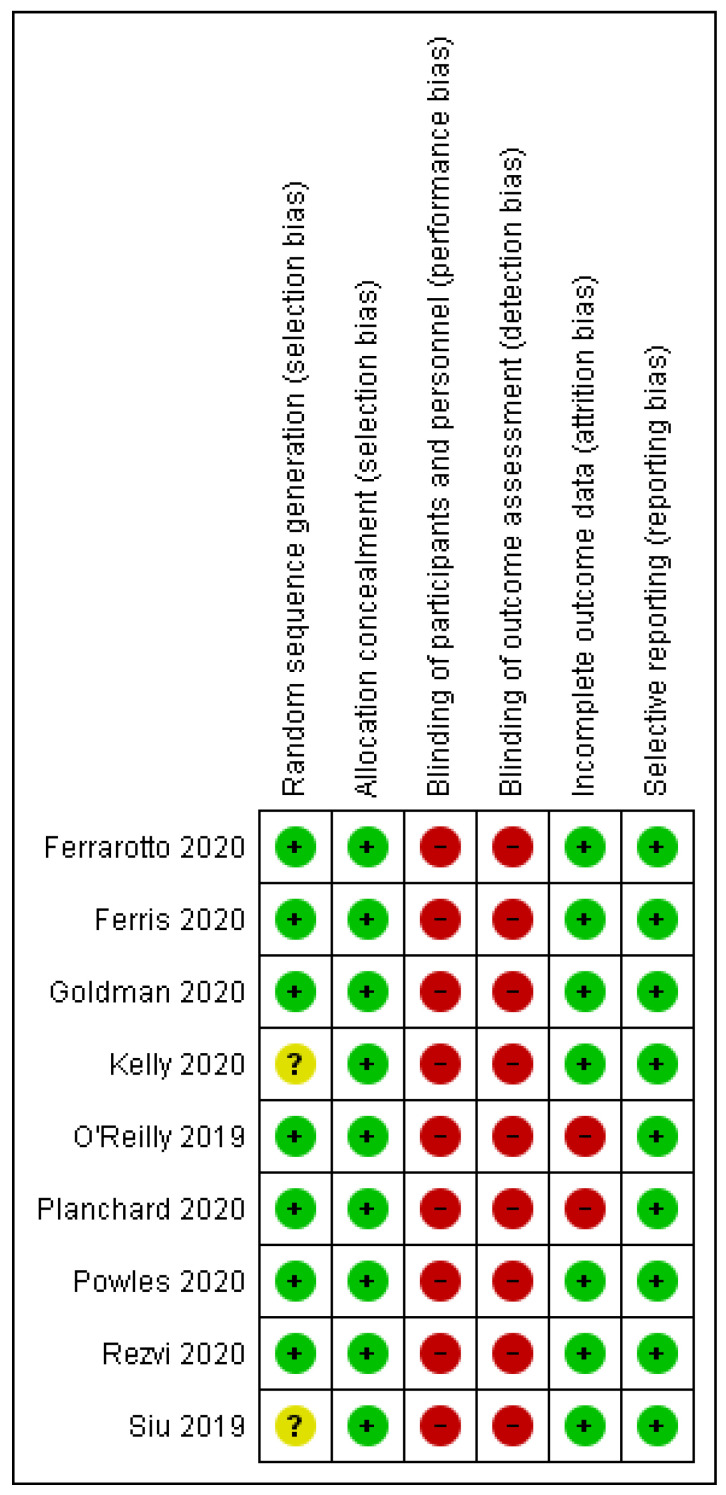
Risk of bias in the randomized trials: green, low risk; yellow, unclear; red, high risk.

Risk of bias assessment of the included studies is available in Figure 2 and Figure 3.

### 3.2. Overall Incidence of Side Effects

**Figure 4:**
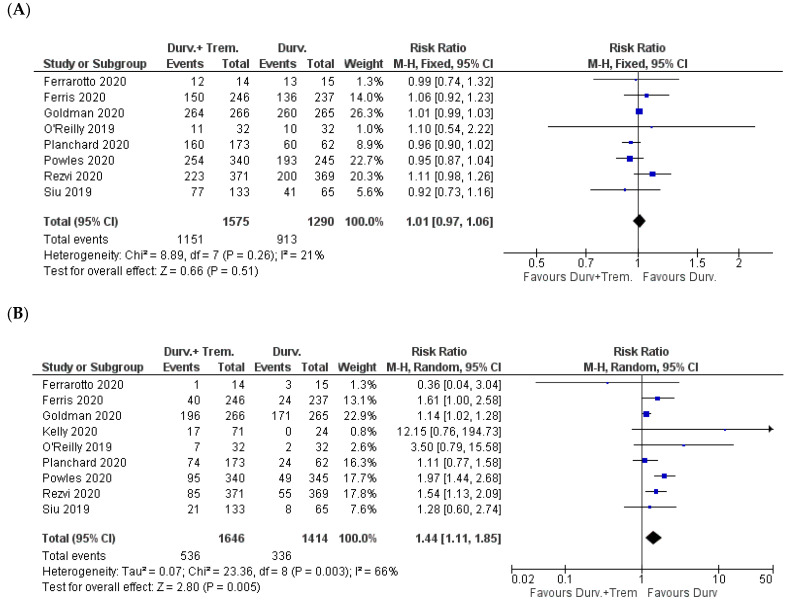
Forest plots for the risk ratio of AEs: (**A**) all AEs; (**B**) AEs ≥ Grade 3.

Considering eight studies with a total of 2865 patients, there was no significant difference in the total number of AEs from any grade. The incidence was 73% (1151/1575) and 70.8% (913/1290) in combination (durvalumab + tremelimumab) vs. monotherapy (durvalumab) patients, respectively (Z = 0.66; *p* = 0.51; RR = 1.01) [22,23,24,26,27,30,31,32] (Figure 4A).

In the comparison of AEs ≥ Grade 3, which included all the studies, the incidence was significantly higher in the combination vs. monotherapy patients: 32.6% (536/1646) vs. 23.8% (336/1414) (Z = 2.80; *p* = 0.005; RR = 1.44) (Figure 4B).

### 3.3. Gastrointestinal Side Effects

**Figure 5:**
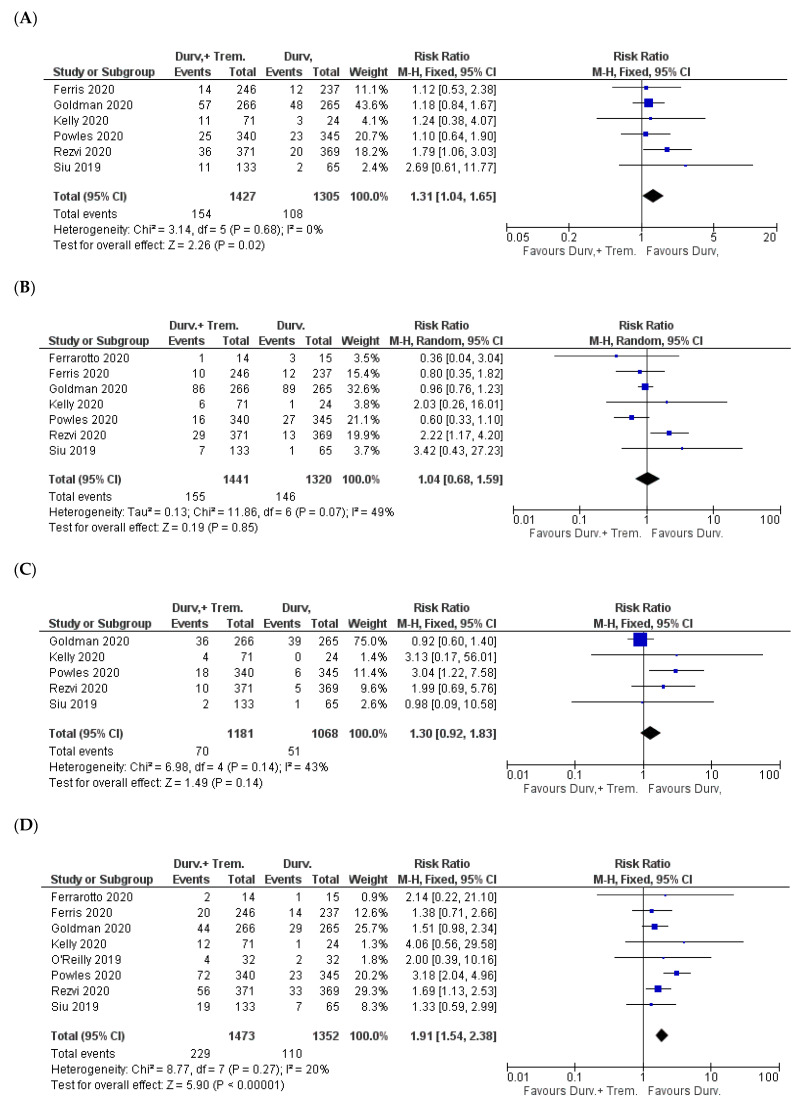

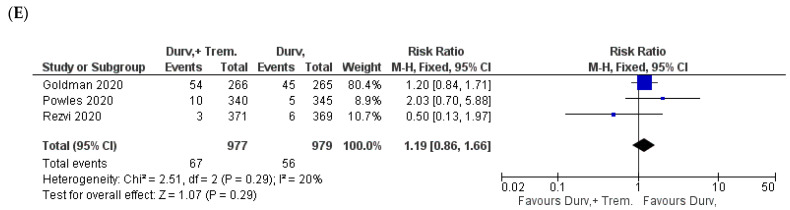
Forest plots for the risk ratio of (**A**) reduced appetite, (**B**) nausea, (**C**) vomiting, (**D**) diarrhea, and (**E**) constipation.

Pooled analysis was feasible for five symptoms (reduced appetite, nausea, vomiting, diarrhea, and constipation). A significantly higher incidence of reduced appetite and diarrhea and a trend toward a higher incidence of vomiting were observed for combination vs. monotherapy patients. For reduced appetite, the incidence was 10.8% (154/1427) in combination vs. 8.3% (108/1305) in monotherapy patients (Z = 2.26; *p* = 0.02; RR = 1.31) [23,24,25,30,31,32] (Figure 5A).

Nausea was reported to be 10.8% (155/1441) in combination vs. 11% (146/1320) in monotherapy patients (Z = 0.19; *p* = 0.85; RR = 1.04) [22,23,24,25,30,31,32] (Figure 5B). For vomiting, the incidence was 5.9% (70/1181) in combination vs. 4.8% in monotherapy (51/1068) (Z = 1.49; *p* = 0.14; RR = 1.30) [24,25,30,31,32] (Figure 5C). Diarrhea was reported in 15.6% (229/1473) in combination vs. 8.1% in monotherapy (110/1352) (Z = 5.90; *p* < 0.00001; RR = 1.91) [22,23,24,25,26,30,31,32] (Figure 5D). Constipation was reported in 6.9% (67/977) in combination vs. 5.7% (56/979) in monotherapy (Z = 1.07; *p* = 0.29; RR = 1.19) [24,30,31] (Figure 5E).

### 3.4. Dermal Manifestations

**Figure 6:**
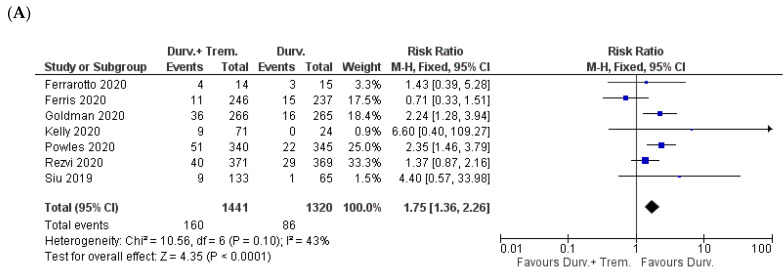

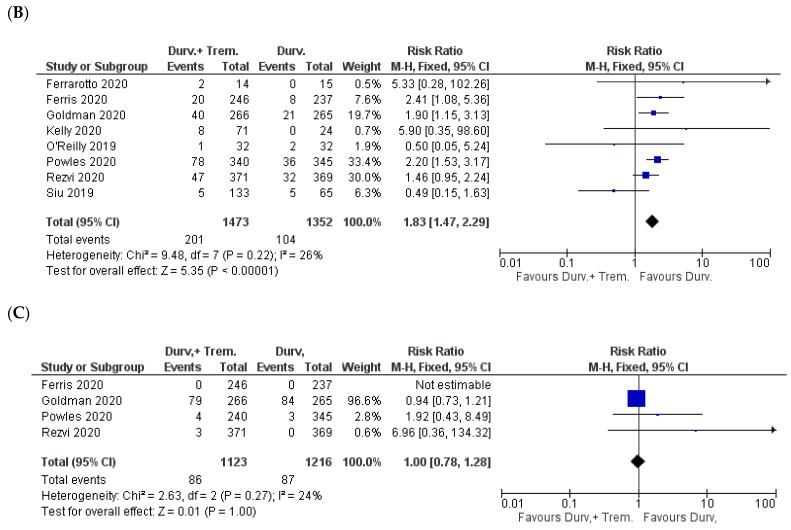
Forest plots for the risk ratio of (**A**) rash, (**B**) pruritis, and (**C**) alopecia.

With regard to dermal manifestations, rash, pruritis, and alopecia were considered and compared. The incidence of both rash and pruritis was significantly higher in combination vs. monotherapy patients. As shown in Figure 6A, rash incidence was 11.1% (160/1441) in combination vs. 6.5% (86/1320) in monotherapy (Z = 4.35; *p* < 0.0001; RR = 1.75) [22,23,24,25,30,31,32].

For pruritis, the incidence was 13.6% (201/1473) in combination vs. 7.7% (104/1352) in monotherapy patients (Z = 5.35; *p* < 0.00001; RR = 1.83) [22,23,24,25,26,30,31,32] (Figure 6B). Comparable incidence was reported for alopecia, which was 7.7% (86/1123) in combination vs. 7.2% (87/1216) in monotherapy (Z = 0.01; *p* = 1.00; RR = 1.00) [23,24,30,31] (Figure 6C).

### 3.5. Hematological Side Effects

**Figure 7:**
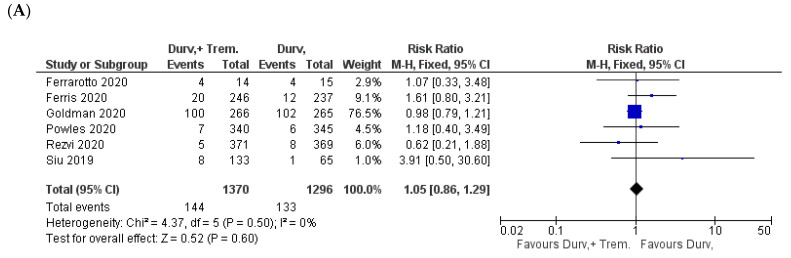

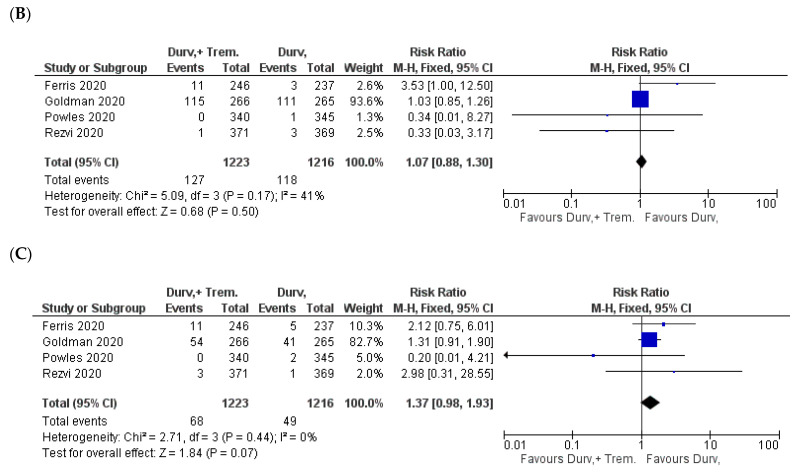
Forest plots for the risk ratio of (**A**) anemia, (**B**) neutropenia, and (**C**) thrombocytopenia.

When considering hematological side effects, no significant difference in the incidence of anemia, neutropenia, and thrombocytopenia was observed. However, there was a trend toward a higher incidence of thrombocytopenia for combination vs. monotherapy patients. Anemia was recorded in 10.5% (144/1370) in combination vs. 10.3% (133/1296) in monotherapy (Z = 0.52; *p* = 0.60; RR = 1.05) [22,23,24,30,31,32] (Figure 7A).

Similarly, neutropenia was reported in 10.4% (127/1223) in combination vs. 9.7% (118/1216) in monotherapy patients (Z = 0.68; *p* = 0.50; RR = 1.07) [23,24,30,31] (Figure 7B). Lastly, thrombocytopenia was reported in 5.6% (68/1223) in combination vs. 4% (49/1216) in monotherapy (Z = 1.84; *p* = 0.07; RR = 1.37) [23,24,30,31] (Figure 7C).

### 3.6. Metabolic and Endocrine Manifestations

**Figure 8:**
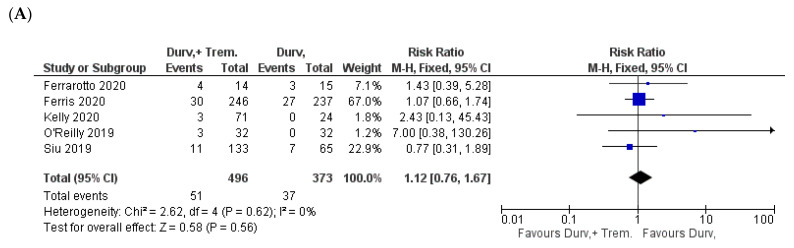

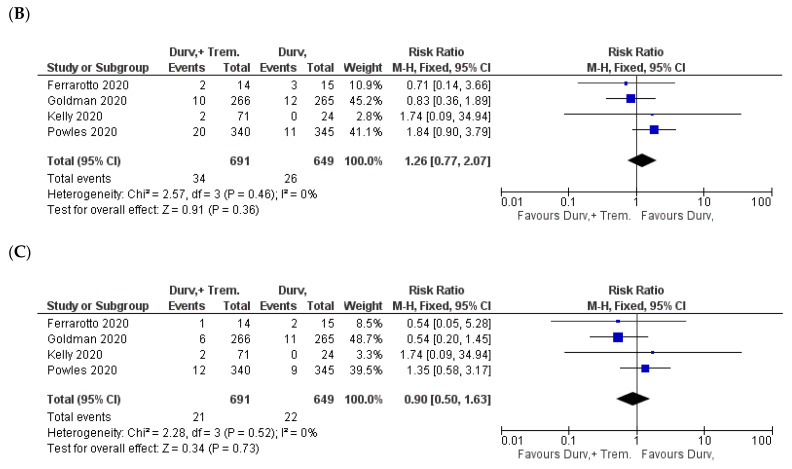
Forest plots for the risk ratio of (**A**) hypothyroidism, (**B**) increased lipase, and (**C**) increased amylase.

With respect to metabolic and endocrine manifestations, a comparable incidence of hypothyroidism as well as an elevation of both lipase and amylase enzymes were observed. Hypothyroidism was reported in 10.3% (51/496) in combination vs. 9.9% (37/373) in monotherapy patients (Z = 0.58; *p* = 0.56; RR = 1.12) [22,23,25,26,32] (Figure 8A).

Increased lipase incidence was 4.9% (34/691) in combination vs. 4% (26/649) in monotherapy patients (Z = 0.91; *p* = 0.36; RR = 1.26) [22,24,25,30]. However, increased amylase incidence was 3% (21/691) in combination vs. 3.4% (22/649) in monotherapy (Z = 0.34; *p* = 0.73; RR = 0.90) [22,24,25,30] (Figure 8B,C).

### 3.7. General Manifestations

**Figure 9:**
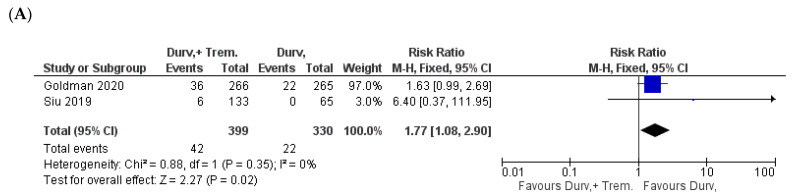

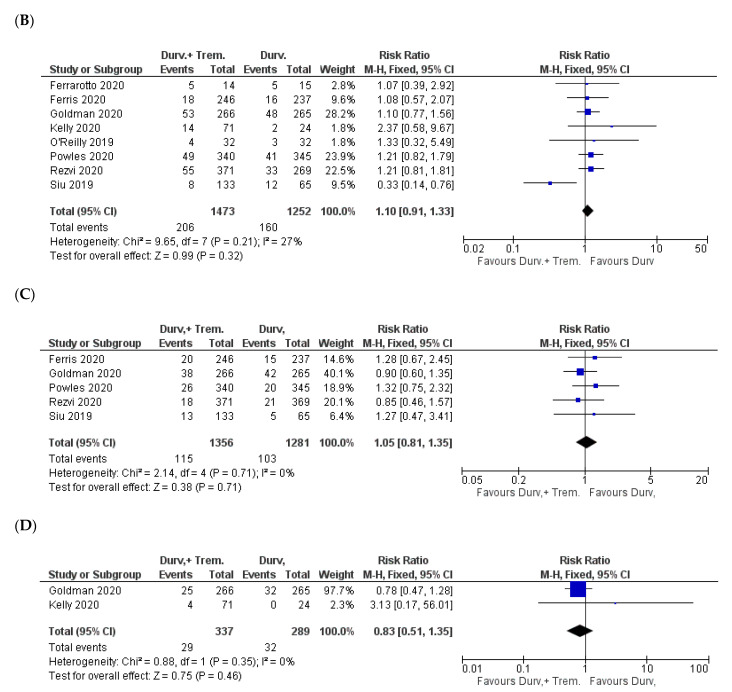
Forest plots for the risk ratio of (**A**) fever, (**B**) fatigue, (**C**) asthenia, and (**D**) dyspnea.

Among fever, fatigue, asthenia, and dyspnea, only fever showed significant higher incidence in combination vs. monotherapy patients. Fever was 10.5% (42/399) in combination vs. 6.6% (22/330) in monotherapy (Z = 2.27; *p* = 0.02; RR = 1.77) [24,32] (Figure 9A).

Fatigue was reported in 14% (206/1473) in combination vs. 12.8% (160/1252) in monotherapy patients (Z = 0.99; *p* = 0.32; RR = 1.10) [22,23,24,25,26,30,31,32] (Figure 9B). In the case of asthenia, the incidence was 8.5% (115/1356) in combination vs. 8% (103/1281) in monotherapy (Z = 0.38; *p* = 0.71; RR = 1.05) [23,24,30,31,32] (Figure 9C). Dyspnea was insignificantly lower in combination therapy, 8.6% (29/337) vs. 11% (32/289) in monotherapy (Z = 0.75; *p* = 0.46; RR = 0.83) [24,25] (Figure 9D).

### 3.8. Treatment Discontinuation and Mortality

**Figure 10:**
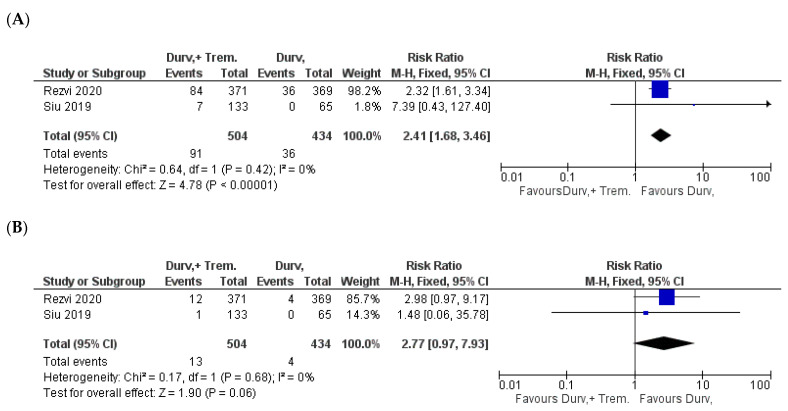
Forest plots for the risk ratio of (**A**) discontinuation and (**B**) death.

Discontinuation rate was markedly higher in combination compared with monotherapy patients; in fact, it was 18% (91/504) in combination vs. 8.3% (36/434) in monotherapy (Z = 4.78; *p* < 0.00001; RR = 2.41) [31,32] (Figure 10A).

Mortality for combination vs. monotherapy was also higher, even though with marginal *p*-value, just below the significance level. In particular, death rate was 2.6% (13/504) for combination compared with 0.7% (3/434) for monotherapy patients (Z = 1.90; *p* = 0.06; RR = 2.77) [31,32] (Figure 10B).

**Figure 11:**
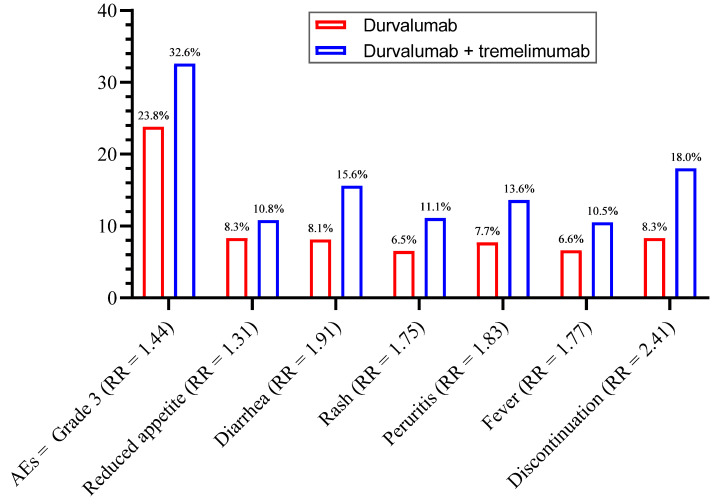
Summary of the significant results (*p* < 0.05).

All significant results of this meta-analysis are summarized in Figure 11.

## 4. Discussion

ICIs are part of the standard of care for patients with many advanced solid tumors, displaying a durable response up to complete regression of metastatic lesions in different cancer types such as NSCLC [33]. One of the challenging points regarding the use of ICIs is represented by the identification of real predictive biomarkers that can help in the selection of patients before starting treatment. In fact, the identification of biomarkers is usually obtained by analyzing tissue biopsies that might not be available for every patient. Especially in the case of patients presenting severe and/or steroid-refractory irAEs, a biopsy sample should be obtained and analyzed for infiltrating immune cells, allowing the selection of novel biological agents targeting crucial inflammatory mediators [11,34]. The difficulty in obtaining biopsies, along with a long time of preservation and technical processing, could lead to the alteration of the molecular properties of the tissue samples [34,35].

ICIs such as durvalumab, tremelimumab, and ipilimumab have been investigated as monotherapy, as well as in different combination approaches, such as with chemotherapy or other ICI agents [36,37,38]. The rationale behind the use of durvalumab and tremelimumab combination is to enhance antitumor immune activity through two different mechanisms related to the inhibition of PD-L1/programmed cell death-1 (PD-1) and CTLA-4 pathways: anti-PD-L1/anti-PD-1 operates in the tumor microenvironment and prevents T cell function inhibition, whilst anti-CTLA-4 acts in the lymphoid compartment to increase the number of tumor-reactive T cells [39,40]. The combination of CTLA-4 and PD-1 blockade has been shown to be able to improve antitumor responses; in this regard, it has been shown that the monoclonal anti-CTLA4 antibody ipilimumab is able to increase tumor-infiltrating T cells and up-regulate the PD-1/PD-L1 inhibitory pathway in a compensatory manner, highlighting how drug combination therapy applications may be very effective [41].

Combined PD-L1 and CTLA-4 inhibition has shown synergistic effects in preclinical models [42], and it has been approved as a first-line therapy for metastatic NSCLC [31], but an open question remains concerning the additional clinical benefit of this combination compared with chemotherapy plus PD-L1 inhibition [43]. Recent studies suggest that patients with metastatic melanoma who progress on PD-L1-directed therapy can respond to combined PD-L1 and CTLA-4 inhibition [44], but it is still not clear the clinical impact of this combination in terms of safety. A very recent study from Schoenfeld et al. [45] found that combined PD-L1 and CTLA-4 inhibition in NSCLC resistant to PD-L1 inhibition was relatively well tolerated, with an overall prevalence of grade 3 or higher treatment-related AEs compared with the 22% prevalence detected with durvalumab–tremelimumab in the ARCTIC trial [27].

In the present systematic review and meta-analysis, we managed to provide a detailed evaluation of the additional risk in terms of safety when combining tremelimumab with durvalumab. It is well-known that the administration of multiple medications could increase the chances of side effects or drug–drug interactions in patients, even though the negative outcome can be tolerable without significant impact on patient life [46]. In the case of the combination of durvalumab and tremelimumab, the current literature does not provide a clear answer on how significant is the risk of combining these two drugs. Addressing this question is very useful to help during counseling of patients before enrollment in such studies as well as to plan future clinical trials. All trials included in this analysis were randomized and controlled, strengthening the level of evidence of our results (libguides.winona.edu/ebptoolkit/Levels-Evidence (accessed on 14 November 2021)). It is worth mentioning that non-randomized controlled trials have not been excluded a priori; however, our research, by using electronic databases, did not lead to the identification of any of them. Since the aim of our study was to investigate the side effects and tolerability, the oncological response was not included in the analysis, as patients had different types of cancers. Therefore, we could not perform a pooled analysis to investigate the efficacy.

Assessment of additional risk should be evaluated by looking at both RR (the probability of having the event) and the absolute risk (the real reported difference in the incidence between the two cohorts) (www.ncbi.nlm.nih.gov/books/NBK63647, (accessed on 27 February 2022)). The highest RR in our study was observed for mortality (RR = 2.77), which means that adding tremelimumab to durvalumab will increase the risk of mortality almost three times when compared with durvalumab administrated alone; however, the *p*-value did not reach the significance level probably due to the low number of studies. A very low incidence of mortality in both arms (2.6% vs. 0.7%) was observed, meaning that the absolute risk of mortality is less than 2%. The second highest risk was identified to be discontinuity, which was about 2.5 folds higher for combination vs. monotherapy. The remaining significant RRs in our analysis were always below 2, meaning that the additional risk coming from the use of tremelimumab with durvalumab to produce those side effects is below two-fold.

As mentioned earlier, more side effects are expected to be reported with combination therapy compared with monotherapy. However, in view of the overwhelming number of reported side effects for ICIs, it is clinically very useful to specify which AEs are more expected when administering a combination therapy. Among the different AEs reported in the included studies, our analysis was able to identify certain AEs, such as fever, diarrhea, rash, pruritis, and reduced appetite, to be higher with combination therapy compared with durvalumab monotherapy. Additionally, a higher risk of treatment discontinuation and mortality was observed.

The results of our analysis point out the fact that especially high-risk patients receiving an ICIs-based therapy should be regularly and frequently monitored for treatment-related complications and, in the best scenario, be subjected to a personalized surveillance strategy [11]. The latter is of utmost importance in light of the fact that the frequency and variability of irAEs could be affected by different variables such as the agents used, the exposure time and the administered dose, and the patient’s intrinsic risk factors.

Despite the high level of evidence presented in this study, there are some limitations that should be considered, for instance, the limited number of included studies. Additionally, some trials are phase I or II. Patients taken into account in the present study had different types of cancers and patient intrinsic risk factors, with or without previous treatments, which may affect the outcome. Lastly, in some studies, patients received chemotherapy along with immunotherapy. The variation in immunotherapy protocols, drug doses, the duration of therapy, and follow-up might have impacted the tolerability. All these factors will limit the clinical impact of this study. Recent studies suggest that durvalumab–tremelimumab combination can provide meaningful clinical benefit in specific tumors such as the subgroup of patients with NSCLC who progressed on PD-1-directed therapy [45], but it will be essential in future studies to assess whether biomarkers of tumor-infiltrating CD8+ and CD4+ T cells at baseline are associated with an increased response to combined PD-L1 and CTLA-4 inhibition and better tolerability of this combination.

## 5. Conclusions

Our results highlight how the combination of durvalumab with tremelimumab increases the risk of treatment discontinuation and mortality compared with durvalumab monotherapy. Additionally, a higher risk of developing fever, diarrhea, rash, and pruritis, along with reduced appetite, was observed. This must be highlighted to patients during counseling before enrollment in clinical trials in which a combined therapy consisting of durvalumab and tremelimumab will be used. Based on the present study, further investigations, especially regarding safety, are required to justify the use of this drug combination.

## Figures and Tables

**Table 1:** Summary of the included studies.

Study	NCT ID/Trial Name	Phase and Status	Timeframe	Patient Criteria	Sample Size (D + T vs. D)	Doses	Outcome
Ferrarotto 2020 [22]	NCT03144778 (CIAO trial)	Phase I, randomized, open-label, single institution	Jul. 2017–Feb. 2019	Newly diagnosed stage II-IVA oropharynx cancer or locoregionally recurrent oropharynx cancer amenable to resection	14 15	Two cycles of intravenous D 1500 mg ± T 75 mg on day 1 of a 28-day cycle	D + T did not increase CD8+ TIL density more than D alone
Ferris 2020 [23]	NCT02369874 (EAGLE)	Phase III, randomized, open label, multicenter	Nov. 2015–Jul. 2017	Recurrent or metastatic head and neck squamous cell carcinoma	246 236	D (10 mg/kg every 2 weeks (q2w)), D + T (D 20 mg/kg every 4 weeks (q4w) + T 1 mg/kg q4w up to four doses, followed by D 10 mg/kg q2w)	Combining D with T did not show improvement over D activity
Goldman 2020 [24]	NCT03043872 (CASPIAN)	Phase III, randomized, open label, multicenter	Mar. 2017–May. 2018	Treatment-naive, histologically or cytologically documented extensive-stage small-cell lung cancer	266 265	Patients in the immunotherapy groups received four cycles of platinum–etoposide + D 1500 mg ± T 75 mg every 3 weeks, followed by maintenance D 1500 mg every 4 weeks. Patients in the D + T + platinum–etoposide group received one additional dose of T 75 mg after platinum–etoposide (up to five doses)	Addition of T to D plus platinum–etoposide did not significantly improve outcomes vs. platinum–etoposide
Kelly 2020 [25]	NCT02340975	Phase 1b/II, randomized, open label, multicenter	Mar. 2015–Jan. 2018	Metastatic/recurrent gastric or gastroesophageal junction cancer	71 24	D 20 mg/kg + T 1 mg/kg Q4W for four cycles, followed by D 10 mg/kg Q2W. Patients in arm B received D monotherapy (10 mg/kg) Q2W	Response rates were low regardless of monotherapy or combination strategies
Planchard 2020 [27]	NCT02352948 (ARCTIC)	Phase III, randomized, open label, multicenter	Jan. 2015–Sep. 2016	Metastatic NSCLC	173 62	D + T (12 weeks D 20 mg/kg) + T 1 mg/kg q4w then 34 weeks vs. D 10 mg/kg q2w D (up to 12 months 10 mg/kg every 2 weeks (q2w))	The efficacy of D + T was comparable with that of D monotherapy, suggesting a limited contribution of T
Powles 2020 [30]	NCT02516241 (DANUBE)	Phase III, randomized, open label, multicenter	Nov. 2015–Mar. 2017	Untreated patients with unresectable, locally advanced, or metastatic urothelial carcinoma	340 345	D monotherapy (at a fixed dose of 1500 mg, administered intravenously every 4 weeks); the combination of D (1500 mg) and T (75 mg), both administered intravenously every 4 weeks for up to four doses, followed by D maintenance monotherapy (1500 mg, administered intravenously every 4 weeks)	Combination treatment suggests that T has activity in this disease when given in combination with D, but it also increases toxicity
Rezvi 2020 [31]	NCT02453282 (MYSTIC)	Phase III, randomized, open label, multicenter	Jul. 2015–Jun. 2016	Metastatic NSCLC	371 369	D (20 mg/kg every 4 weeks) plus T (1 mg/kg every 4 weeks, up to 4 doses), D (20 mg/kg every 4 weeks)	D + T combination was associated with a higher rate of AEs, leading to discontinuation of D
O’Reilly 2019 [26]	NCT02558894	Phase II, randomized, open label, multicenter	Nov. 2015–Mar. 2017	Metastatic pancreatic ductal adenocarcinoma	32 32	D therapy (1500 mg every 4 weeks) plus T therapy (75 mg every 4 weeks) for 4 cycles followed by D therapy (1500 mg every 4 weeks) or D monotherapy (1500 mg every 4 weeks) for up to 12 months	The observed efficacy of D + T therapy and D monotherapy was reflective of a population of patients with mPDAC who had poor prognoses and rapidly progressing disease
Siu 2019 [32]	NCT02319044 (CONDOR)	Phase II, randomized, open label, multicenter	Apr. 2015–Mar. 2016	Patients with PD-L1–low/negative recurrent or metastatic head and neck squamous cell carcinoma	133 65	D (20 mg/kg every 4 weeks) + T (1 mg/kg every 4 weeks) for four cycles, followed by D (10 mg/kg every 2 weeks), or D (10 mg/kg every 2 weeks) monotherapy, or T (10 mg/kg every 4 weeks for seven doses then every 12 weeks for two doses) monotherapy	Minimal observed difference between D and D + T

## Data Availability

Not applicable.
